# Analysis of stomatal characteristics of maize hybrids and their parental inbred lines during critical reproductive periods

**DOI:** 10.3389/fpls.2024.1442686

**Published:** 2025-01-16

**Authors:** Changyu Zhang, Yu Jin, Jinglu Wang, Ying Zhang, Yanxin Zhao, Xianju Lu, Wei Song, Xinyu Guo

**Affiliations:** ^1^ Beijing Key Lab of Digital Plant, Information Technology Research Center, Beijing Academy of Agriculture and Forestry Sciences, Beijing, China; ^2^ National Engineering Research Center for Information Technology in Agriculture, Beijing Academy of Agriculture and Forestry Sciences, Beijing, China; ^3^ Beijing Key Laboratory of Maize DeoxyriboNucleic Acid (DNA) Fingerprinting and Molecular Breeding, Maize Research Center, Beijing Academy of Agriculture and Forestry Sciences, Beijing, China; ^4^ Key Laboratory of Crop Genetics and Breeding of Hebei Province, Institute of Cereal and Oil Crops, Hebei Academy of Agriculture and Forestry Sciences, Shijiazhuang, China

**Keywords:** maize, hybrids, stomatal phenotypes, high-throughput acquisition, deep learning

## Abstract

The stomatal phenotype is a crucial microscopic characteristic of the leaf surface, and modulating the stomata of maize leaves can enhance photosynthetic carbon assimilation and water use efficiency, thereby playing a vital role in maize yield formation. The evolving imaging and image processing technologies offer effective tools for precise analysis of stomatal phenotypes. This study employed Jingnongke 728 and its parental inbred to capture stomatal images from various leaf positions and abaxial surfaces during key reproductive stages using rapid scanning electron microscopy. We uesd a target detection and image segmentation approach based on YOLOv5s and Unet to efficiently obtain 11 phenotypic traits encompassing stomatal count, shape, and distribution. Manual validation revealed high detection accuracies for stomatal density, width, and length, with R2 values of 0.92, 0.97, and 0.95, respectively. Phenotypic analyses indicated a significant positive correlation between stomatal density and the percentage of guard cells and pore area (r=0.36), and a negative correlation with stomatal area and subsidiary cell area (r=-0.34 and -0.46). Additionally, stomatal traits exhibited notable variations with reproductive stages and leaf layers. Specifically, at the monocot scale, stomatal density increased from 74.35 to 87.19 Counts/mm2 from lower to upper leaf layers. Concurrently, the stomatal shape shifted from sub-circular (stomatal roundness = 0.64) to narrow and elongated (stomatal roundness = 0.63). Throughout the growth cycle, stomatal density remained stable during vegetative growth, decreased during reproductive growth with smaller size and narrower shape, and continued to decline while increasing in size and tending towards a rounded shape during senescence. Remarkably, hybrid 728 differed notably from its parents in stomatal phenotype, particularly during senescence. Moreover, the stomatal density of the hybrids showed negative super parental heterosis (heterosis rate = -0.09), whereas stomatal dimensions exhibited positive super parental heterosis, generally resembling the parent MC01. This investigation unveils the dynamic variations in maize stomatal phenotypes, bolstering genetic analyses and targeted improvements in maize, and presenting a novel technological instrument for plant phenotype studies.

## 1 Introduction

Maize, as one of the world’s most important food crop, the improvement of its yield and traits has been a central goal of breeding research (Tollenaar and Lee, 2002; Lobell et al., 2014). In the process of achieving high, stable, high-quality and safety as well as enhanced stress tolerance, the leaf plays a crucial role as the main site of photosynthesis and material metabolism (Huang et al., 2017). The stomata located in the epidermis of leaves, which are composed of guard cells, subsidiary cells and pores, are the key channels for gas exchange and water regulation between plants and the external environment (During, 1980; Hui et al., 2003). The characteristics of stomata, including number, size, morphology and distribution, directly affect the physiological function and growth and development of maize. Among them, stomatal pores, as direct channels for water-gas exchange, are precisely regulated by the size and opening and closing states of the guard cells (Bertolino et al., 2019), while the subsidiary cells provide the necessary support and stability for the guard cells. The formation and variation of these stomatal characteristics are not only determined by genetic factors, but also profoundly influenced by the environmental conditions of growth.

As fertility advances, stomatal characteristics undergo a series of changes to adapt to different growth environments and developmental requirements, and external signals perceived by mature leaves also regulate stomatal development on the epidermis of new leaves, leading to changes in stomatal patterns (Zarinkamar, 2007). Ji et al. (2008) found that there are significant differences in leaf stomatal characteristics of maize varieties during the whole reproductive period, with the advancement of fertility leaf stomatal density gradually increased, leaf stomatal area decreased significantly, leaf stomatal length and stomatal width first increased and then decreased. The differences in leaf stomatal characteristics of different maize varieties are related to leaf position. Stomata in different parts of the leaf, the stomatal density varies greatly. Generally stomatal density increases gradually from the midvein portion of the leaf blade to the edge of the leaf blade ^[11]^. Salisbury considered the gradual increase in stomatal density from the base to the top of the plant and the high stomatal density of leaves borne high up as one of the characteristics of the plant, independent of the area of a single leaf (Salisbury, 1928). A fairly stable negative correlation was found between stomatal size and stomatal number (Blanke, 1994; Franks et al., 2009). The ratio of open and closed stomata is related to their resistance under stress conditions (Bertolino et al., 2019), and stomatal pore opening is directly related to CO_2_ uptake (Toh et al., 2021). Therefore, in order to study the pattern of stomatal phenotypic characteristics and their relationship with maize growth and development more deeply, it is necessary to flexibly utilize various phenotypic acquisition and resolution methods.

In recent years, with the rapid development of microscopy techniques and computer vision algorithms, breakthroughs have been made in the methods of acquiring and resolving stomatal phenotypes. In traditional methods, stomatal observation often requires destructive sampling, such as the fresh-sample method or the imprint method, which is performed by obtaining leaf epidermal strips (Zelitch, 1969; Weyers and Travis, 1981) or leaf surface imprints (Horanic and Gardner, 1967; Witham et al., 1971) for observation under a bright-field microscope. However, these methods have limitations that prevent real-time and high-throughput observations. With the continuous advancement of technology, non-destructive sampling methods have gradually come to the fore. *In situ* observation methods and optical topography measurements are capable of obtaining real-time information on leaf stomatal surfaces. These methods use high-precision mobile micro-imaging equipment to acquire stomatal phenotypes, and although the imaging efficiency is high, the image quality and sample size are still limited, preventing the realization of truly high-throughput observations. Optical topography measurements, on the other hand, achieves high-resolution and high-throughput image acquisition without the need for sample preparation, but its fabrication process is relatively cumbersome.

In the early stage of stomatal phenotyping, we mainly relied on manual methods (Long and Clements, 1934; Kubínová, 1994), such as using microscope to measure stomatal dimensions on a microscale, recording the number of stomata through counting chambers and Veeder-Root, and obtaining stomatal density through the sample strip method. These methods are not only time-consuming and labor-intensive, but also limited in the amount of stomatal phenotypic traits and data obtained, which cannot meet the needs of large-scale experiments. With the continuous development of precision microscopic instruments and techniques, nondestructive observation of stomata has become possible (Wu et al., 2009; He et al., 2019; Liang et al., 2022), and the means of analyzing stomatal phenotypes are becoming simpler, more automated and more intelligent. The emergence of semi-automated and automated software has greatly improved the efficiency of stomatal phenotype analysis. The wide application of deep learning and other techniques in feature parsing makes the acquired stomatal phenotypic traits more perfect and the parsing of phenotypic feature differences more in-depth (Vialet-Chabrand and Brendel, 2014; Li et al., 2019). Semi-automated image-based stomatal parsing has gradually taken shape and various automatically integrated measurement software has emerged, such as Image J, Scope Image9.0 and Motic Images Advance. These software can easily obtain various phenotypic traits of stomata, such as stomatal number, width, length, area, perimeter and shape traits.

The emergence of automated phenotyping tools has solved the bottleneck problem of trait phenotyping, making large-scale, high-throughput stomatal phenotyping studies possible. With the sophistication of measuring instruments and the continuous development of computer vision algorithms, directly observed stomatal data were gradually transformed into image data, resulting in convenient phenotypic trait extraction methods. For example, Bheemanahalli et al. (2021) used deep learning to resolve stomatal density and guard cell characteristics in sorghum, and identified 71 loci (38 environment-specific) genotypes with significant stomatal traits in combination with genome-wide association studies (GWAS). Liang et al. (2022) utilized an improved CV model for stomatal pore size phenotypic trait resolution. In addition, Li et al. (2022) developed LeafNet widely applicable to segmentation of intact stomata and epidermal cells for each microscopic image. In summary, from the traditional destructive sampling and manual observation to the current non-destructive sampling and automated and intelligent parsing, stomatal phenotyping studies are gradually converging towards high throughput, high precision and high efficiency. These technological advances have provided us with powerful tools to explore the relationship between stomatal phenotypes and maize growth and development, genetic breeding, and resistance.

Hybrid dominance, the phenomenon that hybrids outperform their parents in terms of growth potential, vigor, resistance, yield and quality (Birchler, 2003), has been a hot research topic in the fields of genetics and crop breeding. Previous studies on hybrid dominance have mainly focused on the genetic basis and molecular mechanisms of growth and development, resistance, yield and quality (Stuber et al., 1992; Birchler, 2015), while the contribution and mechanism of stomata, an important physiological structure in hybrid dominance, are relatively poorly understood. However, it has been shown that the stomatal morphology of hybrids is usually more narrow and elongated than that of their parental autogamous lines, with stomatal lengths significantly greater than the mean values of the parents, even greater than those of the parents with higher values (Wang et al., 2004). At the same time, leaf stomatal width of the hybrids was slightly lower than the mean value of the parents. These characterization differences reflect the obvious difference in stomatal morphology and size between hybrids and parents. However, the current research on these differences is still obviously insufficient, with relatively single observation traits and weak correlations among the traits. Therefore, in order to understand the stomatal phenotypic differences between hybrids and parents more comprehensively, we need to synthesize the information of multiple indicators and explore them from multiple perspectives in depth.

In order to fill this research gap, the Jingnongke 728 maize hybrid and its parents were selected as research materials in this study (Wang et al., 2017), and high-definition images of stomata in the lower epidermis of leaves of the whole plant at multiple fertility periods were obtained using the rapid scanning electron microscope (RSEM), and the stomatal phenotypes were accurately analyzed by combining target detection and deep learning techniques. By comparing and analyzing the differences in 11 stomatal phenotypes, including stomatal density, shape, and size between the hybrids and their parents, we expect to reveal the patterns of these differences in different fertility periods and leaf layers, and explore the intrinsic links between stomatal phenotypes and hybrid dominance, so as to provide a powerful reference for the genetic analysis of stomatal phenotypes of maize leaves and the directional improvement of traits in the later stage of the genetic analysis.

## 2 Materials and methods

### 2.1 Plant material and growth conditions

The hybrid Jingnongke 728 independently selected by Beijing Academy of Agricultural and Forestry Sciences and its parent Jing 2416 and maternal MC01 were selected as test materials for this experiment, which were planted at the experimental base in the courtyard of Beijing Academy of Agricultural and Forestry Sciences (139°56′N, 116°16′E) on June 26, 2021 and June 15, 2022, respectively, under the soil conditions as described in the paper of (Zhang et al., 2018).The soil condition was as follows. The experiment was conducted in a completely randomized block design, and the maize was planted at a density of 6 plants/m^2^ with a row spacing of 0.6 m. The planting density and water and fertilizer management were based on the local field production pattern.

Sampling was carried out every 7 days from the three-leaf stage at 6-8a.m. A total of nine sampling occasions, with three plants taken as replicates from each plot. The whole plant leaves were stratified according to the differences in plant height during the reproductive period of maize (Guo and Li 1999; Jiang et al., 2005), and were divided into upper, middle and lower leaves. Sample leaves were obtained from the unfolded leaf near the veins of the whole plant of each variety and immediately fixed in FAA solution (90:5:5, 70% ethanol: 100% formaldehyde: 100% acetic acid) for later studies.

### 2.2 Stomatal phenotype acquisition

The fixed leaf samples were taken out and slightly dried, and a sample block with an area size of about 1 cm^2^ was fixed with conductive adhesive on the carrier stage, with the back side of the leaf facing upwards, and pictures of the lower epidermis of the leaf blade were taken using a flyer Rapid Scanning Electron Microscope (RSEM, Phenom Pro, Netherlands) at a magnification of 350x (767 μm × 767 μm) and 900× (298 μm × 298 μm) images of the leaf lower epidermis. The voltage was high resolution (10 kV), the beam intensity was standard beam current, the probe mode was compositional morphology mode, the image pixels were 1024piex × 1024piex, and four to five different fields of view were taken for each leaf (**Supplementary Figure 1**). In the microscopic observation of stomata, images with different magnifications provided different information details. 350× images showed stomata densely arranged in parallel, which better reflected the distribution characteristics of stomata and was suitable for extracting information on the number of stomata; 900× images showed larger stomata, with more prominent guard cells and subsidiary cells, which was suitable for extracting the morphological and structural characteristics of stomata.

#### 2.2.1 Stomatal number extraction

**Figure 1:**
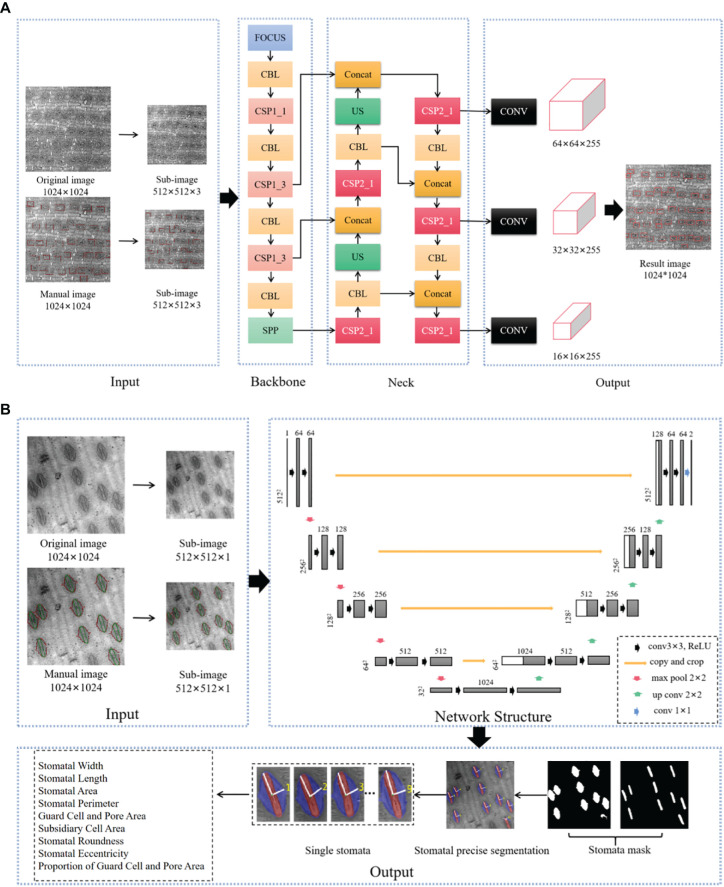
Stomatal Image Recognition and Segmentation Network Architecture Diagram. **(A)**: Input: Adaptive scaling of the image with Mosaic data enhancement to automatically calculate the optimal anchor frame values for the dataset. Backbone: contains the Focus structure and the CSPNet cross-stage localized fusion network. Middle Layer: Contains Path Aggregation Net (PANet) and Space Syramid Pooling (SPP) modules. Output: uses Generalized IoU Loss (GIOU_Loss) as a loss function; **(B)**: The encoder and decoder are basically symmetric, forming a U-shaped network. The encoder is a compression process that consists of four layers, each consisting of two convolutional layers, a maximum pooling layer, and an activation function (Rectified Linear Unit (ReLU)) that doubles the number of eigenchannels after downsampling in each layer. This process reduces the image size by convolution and downsampling and extracts some shallow image features. Decoder is the process of decoding, each layer first uses inverse convolution to double the size of the feature map, and the number of feature channels is halved after inverse convolution. This process acquires some deep image features by deconvolution and upsampling. Jump concatenation is a jump concatenation process, where the feature maps obtained in the encoding and decoding stages are spliced together, combined with deep and shallow features, convolved twice to get more accurate results, and finally classified by 1x1 convolution. Figure from Jin, Y. (2023). Identification of Stomatal Phenotypes in the Ear Leaves of Maize Inbred Lines and Genome-Wide Association Analysis [D]. Huazhong Agricultural University. doi:10.27158/d.cnki.ghznu.2023.001858.


From the 350-fold RSME image set, 420 sheets were randomly selected, and the open-source visual annotation tool labelimg was used to annotate the enclosing frame of each stomatal hole in the image, and the annotated images were subsequently divided into training set, validation set and test set according to the ratio of 7:2:1. The number of stomata was detected using the s-model of YOLOv5 (https://github.com/ultralytics/yolov5). In this study, YOLOv5s, which is the smallest, fastest and flexible of the four YOLOv5 models, was chosen for target detection of stomatal images (**Figure 1A**). The other hyperparameters of training YOLOv5s were set to 2 for batch_size, and the Stochastic Gradient Descent (SGD) optimizer (Rabano et al., 2018) was used, and the network converged after 200 epochs with a momentum factor of 0.86 and a weight decay coefficient of 0.00058. The cosine annealing strategy is used to dynamically adjust the learning rate, which is 0.00258. GIOU_Loss is used as the loss function. The formula is as follows:

(1) $$GIOU\_LOSS=1-GIOU=1-\left(\left|\frac{X\cap Y}{X\cup Y}\right|-\frac{\left|Z-\left(X\cup Y\right)\right|}{\left|Z\right|}\right)$$

Where, X - the set of predicted pixel points, Y - the set of target pixel points, Z - the set of pixel points of the smallest outer rectangle of the prediction and target boxes.

The 1024 × 1024 image was reduced to 512 × 512 and then input into the YOLOv5s model to detect the number of stomata, and then calculate the stomatal density, the number of stomata per unit area of the image (**Figure 1A**). Precision (P) and recall (R) are common metrics commonly used to assess model accuracy (Yang et al., 2020). To balance the accuracy recall, the model precision is evaluated using the Average precision (AP) metric. AP50 refers to the AP measurement when the IoU threshold is 0.5. In this paper, the AP50 is used to evaluate the model precision, and the AP50 reaches 0.96 in the training set and 0.95 in the test set, which means that the model precision is high, the target detection effect of stomatal images is good, and it has good accuracy and feasibility, and can be used for the subsequent extraction of the number of stomata. The formula is as follows:


(2) $$P=\frac{TP}{TP+FN}$$

(3) $$R=\frac{TP}{TP+FP}$$

(4) $$\begin{array}{c} AP50=AP^{\text{IoU}-0.5}=\frac{1}{m}\sum_{i}^{m}Pi\\ =\frac{1}{m}\times P1+\frac{1}{m}\times P2+\ldots +\frac{1}{m}\times Pm=\int^{}P\left(R\right)dR\; \end{array}$$

where, TP – the true target region in the predicted target region, FP - the non-target region in the predicted target region, FN – the target region in the predicted as non-target region, m – the number of prediction frames.

#### 2.2.2 Stomatal morphology and size trait extraction

From the 900 times RSME images, the dataset was constructed by labelme, and the semantic objects of intact stomatal complexes and guard cells and pores in 120 900 images were labeled respectively, and the dataset was expanded to 1200 images, and the training and testing sets were randomly divided according to the ratio of 8:2. The UNet network was used to train the intact stomatal complex and guard cell and pore datasets respectively, with the batch_size set to 3 and the learning rate set to 0.001, and the network converged after 100 epochs of training. The loss function is Dice_Loss, the formula is as follows:


(5) $$Dice\;\_\;Loss=1-2\frac{\left|X\cap Y\right|}{\left|X\right|+\left|Y\right|}$$

Where, X – the set of predicted pixel points, Y – the set of target pixel points.

The 900 images were first downsampled twice, the 1024×1024 images were reduced to 512×512, and the complete stomatal complex mask and the guard cells and pores mask were obtained by UNet segmentation, and then the two were summed together to obtain the further refined stomatal structure mask (**Figure 1B**). Individual stomata were extracted based on the connectivity of the masks, and then 10 phenotypic traits of stomatal size and stomatal shape were calculated. In this paper, we use DICE and IOU to evaluate the accuracy of the UNet network, which are used to measure the degree of similarity between two sets, and are common evaluation metrics for image segmentation tasks, and their calculation formulas are as follows, respectively:


(6) $$DICE=\frac{2\left|X\cap Y\right|}{\left|X\right|+\left|Y\right|}$$

(7) $$IOU=\left|\frac{X\cap Y}{X\cup Y}\right|$$

Where, X – the predicted pixel point set, Y – the target pixel point set.

The final Dice accuracies of stomatal complex and guard cell and pore images on the training set were 0.95 and 0.93, and the IOUs were 0.90 and 0.87, respectively; and the Dice accuracies on the test set were 0.94 and 0.93, and the IOUs were 0.89 and 0.87, respectively, which indicated that the segmentation of stomatal images using UNet was more effective, with a better accuracy and feasibility. The segmentation effect of UNet network is shown in the figure (**Supplementary Figure 2**), which provides strong support for the subsequent extraction and analysis of stomatal phenotype traits.

### 2.3 Phenotypic reliability assessment

One hundred images each were randomly selected at 350x and 900x, and the Digimizer software (https://www.digimizer.com/) was used to manually count the 350x images, measure the stomatal width and stomatal length in the 900x images and fit them to the automated outputs, and calculate the R^2^ and root mean square error (RMSE) for image reliability assessment.

The formula are as follows:


(8) $$R^{2}=1-\frac{\sum_{i}{\left(x_{i}-y_{i}\right)}^{2}}{\sum_{i}{\left(x_{i}-\bar{y_{i}}\right)}^{2}}$$

(9) $$RMSE=\sqrt{\frac{\sum_{i}{\left(x_{i}-y_{i}\right)}^{2}}{n}}$$

Where, *x_i_* – the automated output value, *y_i_* – the manually measured value.

### 2.4 Statistical analysis

The data were initially processed using Microsoft Excel 2013, and R 4.2.1 was used to organize and analyze the data of 11 phenotypic shapes. The acquired phenotypic traits were analyzed from descriptive statistics and the data of each phenotype were interval and scale variables, with some linear relationship, roughly conforming to normal distribution, the data were paired and without outliers, Pearson’s correlation analysis between the indicators was performed, applying ggpairs package and vegan package. ANOVA (Analysis of variance) was done using ggplot2 plotting, Principal components analysis (PCA) between phenotypes and plotting was done using FacoMiner package, ggplot2 package, factoextra package and corrplot package, and the original data were firstly normalized by z-score, and then calculated by calculating the z-score. score normalization, then calculate the correlation coefficient matrix, eigenvalues and eigenvectors. The eigenvalues, the variance of each principal component in the extraction results, were converted to standard deviation. Then the variance contribution ratio and cumulative contribution ratio were calculated to compute the principal component scores, and the data in the graphs are the mean ± standard deviation.

## 3 Results

### 3.1 Analysis of stomatal phenotypic traits and evaluation of accuracy

In this study, the effective fusion of YOLOv5s and UNet network was utilized to realize the automatic extraction of stomatal phenotypic traits. Through the use of deep learning techniques, 11 key stomatal-related traits were successfully obtained, covering multiple dimensions from stomatal number, size to shape (**Table 1**). Among them, one trait of number, six traits of size and four traits of shape were identified.

**Table 1:** Description of stomatal phenotypic traits in maize leaves.

Group	Trait	Abbreviation	Description	Unit
Number	Stomatal density	SD	Number of stomata in unit area	Count/mm^2^
Size	Stomatal width	SW	Width of stomata	um
	Stomatal length	SL	Length of stomata	um
	Stomatal area	SA	Area of individual stomata	um^2^
	Stomatal perimeter	SP	Perimeter of individual stomata	um
	Guard cell and pore area	GCPA	Area of guard cell and pore	um^2^
	Subsidiary cell area	SCA	Area of subsidiary cell	um^2^
Shape	Stomatal roundness	SR	Roundness of individual stomata	–
	Stomatal eccentricity	SE	Eccentricity of individual stomata	–
	Percentage of guard cell and pore area	PGCPA	Guard cell and pore area divided by stomata area	–
	Percentage of subsidiary cell area	PSCA	Subsidiary cell area divided by stomata area	–

**Figure 2:**
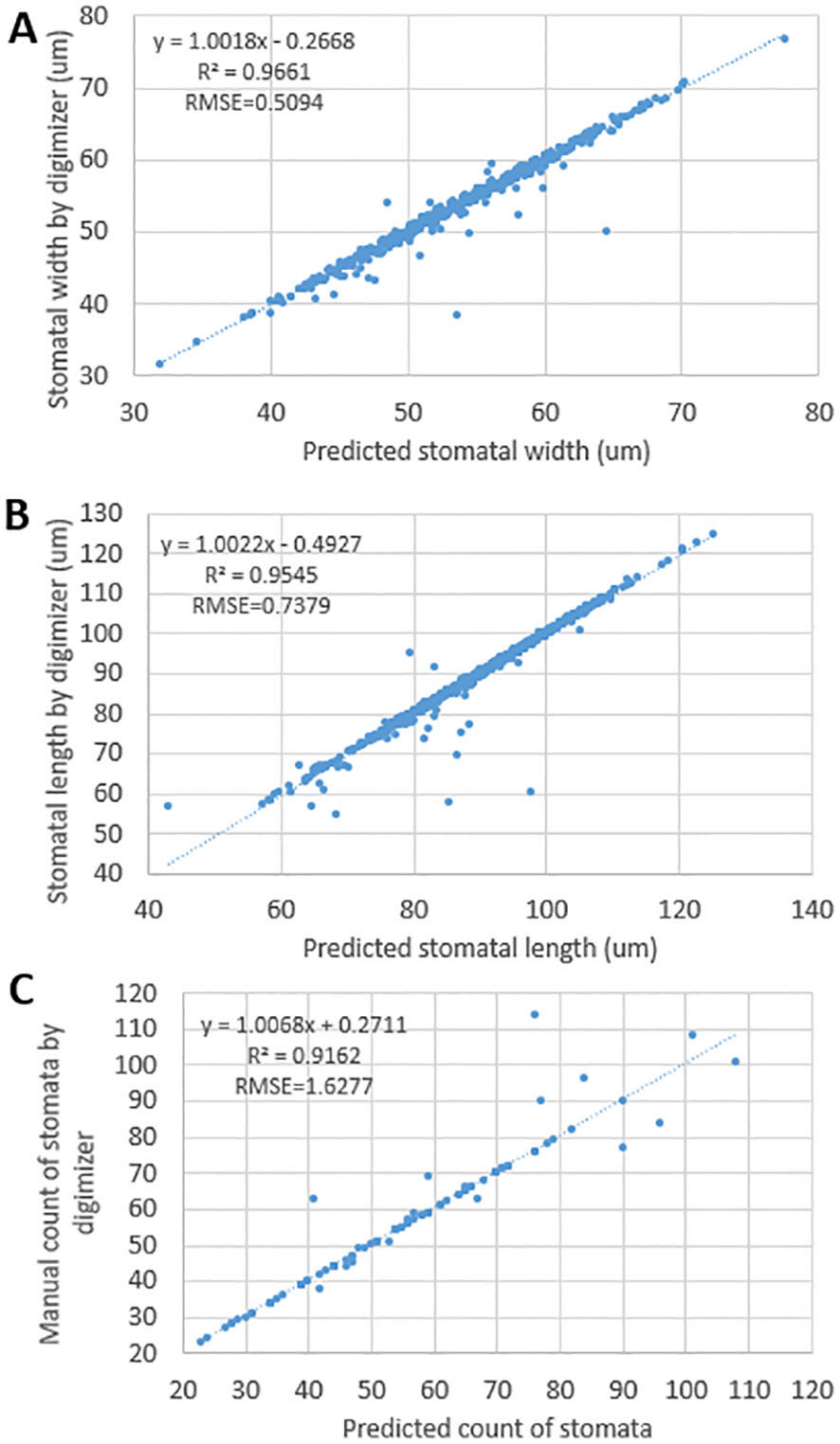
Linear regressions between predicted results and manual results by Digimizer of stomata. **(A)** represents stomatal width, **(B)** represents stomatal length, **(C)** represents stomatal count.

To ensure the accuracy of these automatically extracted traits, three traits, SW, SL and SD, were selected in this study for rigorous comparative analysis with manual measurements, and the results showed that the automated detection and manual measurements showed a high degree of correlation in stomatal width and stomatal length, with correlation coefficients (R²) of 0.97 (**Figure 2A**), respectively, 0.95 (**Figure 2B**) and 0.92 (**Figure 2C**). Meanwhile, the root mean square error (RMSE) was also kept at a low level of 0.51, 0.74 and 1.63, respectively, this result indicates that the automatic stomatal phenotype detection method in this study has high accuracy and reliability.

### 3.2 Correlation analysis of stomatal phenotypic trait

**Figure 3:**
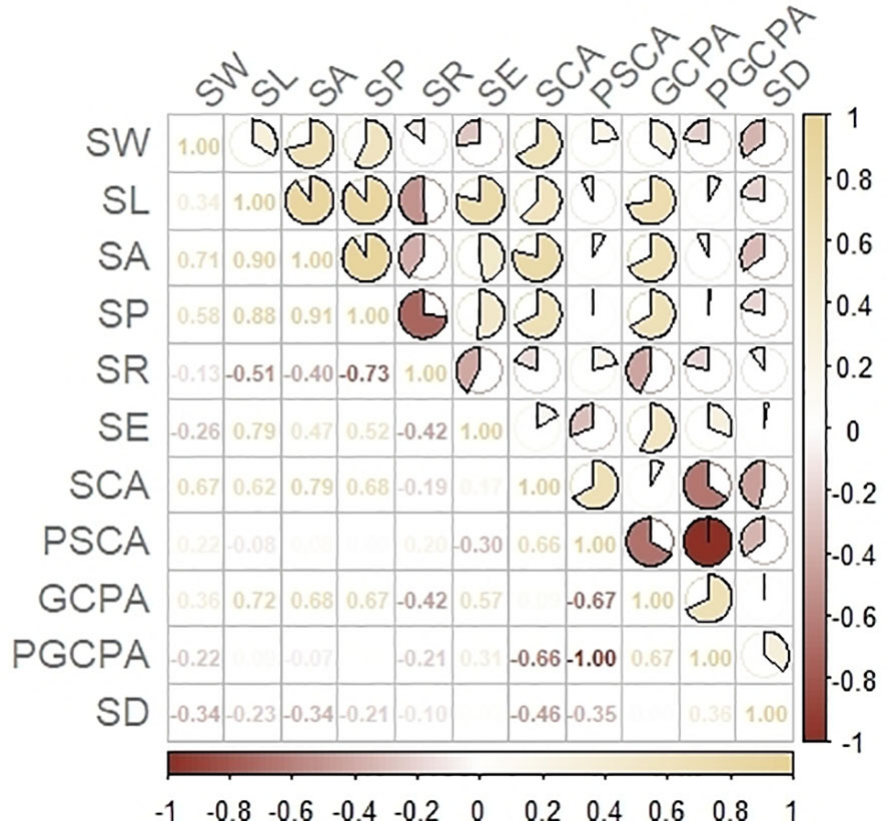
Correlation analysis of stomatal phenotypic traits.

In this study, Pearson correlation analysis was used to analyze the correlation between stomatal phenotypes, and the results showed that there were some correlations between stomatal number, size and shape class traits (**Figure 3**). Among them, SD was significantly positively correlated with the stomatal shape trait PGCPA (r=0.36) and negatively correlated with PSCA (r=-0.35), but had no significant correlation with other shape traits (r>-0.30); it was significantly negatively correlated with the stomatal size traits SA and SCA (r<-0.30), and had no significant correlation with other size traits (r>-0.30).

The correlation coefficients were higher than 0.65 and highly correlated, except for the correlation coefficients between SCA and GCPA, SL and SA, SW and SL, SP and GCPA.

There was a highly significant negative correlation between PSCA and PGCPA (r = -1.0), a significant negative correlation between SR and SE (r = -0.42), and no significant correlation between the rest of the shape traits (r > -0.30).

### 3.3 Dynamic evolution of stomatal phenotypes during different reproductive periods in maize

This study revealed significant differences in stomatal phenotypes of maize during different reproductive periods (**Table 2**). From day 46 to day 110 after sowing, the growth of maize could be clearly divided into three stages: the vegetative growth period (46 to 60 days after sowing), the reproductive growth period (67 to 80 days after sowing) and the aging period (89 to 110 days after sowing). It is worth noting that at the early reproductive growth period from 67 to 73 days after sowing, all stomatal phenotypic traits of the parent 2416, MC01 and the hybrid 728 did not differ significantly among the sampling time points, indicating that there was basically no change in stomatal traits during this period.

**Table 2:** Variance analysis of the effects of date layer and cultivar on stomatal phenotypic characteristics of maize.

		SW	SL	SA	SP	SR	SE	SCA	PSCA	GCPA	PGCPA	SD
Cultivar	2416	55.45 ± 2.24b	90.68 ± 7.15c	3809.86 ± 372.17b	281.48 ± 22.74b	0.61 ± 0.05c	0.77 ± 0.05b	1891.25 ± 267,53b	0.49 ± 0.06b	1919.61 ± 274.43b	0.50 ± 0.06a	87.21 ± 10.97a
	MC01	56.66 ± 4.16a	94.09 ± 8.55b	4053.70 ± 538.99a	281.63 ± 23.36b	0.65 ± 0.04a	0.78 ± 0.04b	2102.48 ± 373.74a	0.52 ± 0.06a	1951.22 ± 318.97ab	0.48 ± 0.06b	78.64 ± 9.41b
	728	55.86 ± 3.25ab	97.20 ± 8.39a	4140.05 ± 464.19a	288.45 ± 22.74a	0.63 ± 0.04b	0.80 ± 0.04a	2122.02 ± 366.05a	0.51 ± 0.06ab	2018.03 ± 287.81a	0.49 ± 0.06ab	74.95 ± 11.82c
Layer	low	56.89 ± 3.56a	90.32 ± 9.89b	3911.35 ± 592.12b	278.74 ± 28.08b	0.64 ± 0.05a	0.76 ± 0.05c	2143.78 ± 377.75a	0.55 ± 0.06a	1767.57 ± 359.52b	0.45 ± 0.06c	74.35 ± 10.86c
	Middle	56.87 ± 2.75a	96.88 ± 5.98a	4178.52 ± 360.10a	291.85 ± 17.73a	0.62 ± 0.04b	0.80 ± 0.03b	2098.11 ± 299.28a	0.50 ± 0.04b	2080.41 ± 137.72a	0.50 ± 0.03b	80.01 ± 8.75b
	Up	53.99 ± 2.68b	96.17 ± 6.82a	3952.73 ± 386.86b	282.99 ± 18.58b	0.63 ± 0.03b	0.82 ± 0.03a	1860.41 ± 306.46b	0.46 ± 0.04c	2098.32 ± 158.02a	0.53 ± 0.04a	87.19 ± 12.21a
Date	46	56.93 ± 3.91a	89.80 ± 9.06def	3877.05 ± 497.27cd	275.21 ± 25.27de	0.65 ± 0.06a	0.75 ± 0.06e	2003.92 ± 307.40bcd	0.52 ± 0.08	1873.13 ± 443.53cd	0.48 ± 0.08	74.43 ± 10.58d
	52	54.58 ± 3.37b	88.56 ± 9.38ef	3696.80 ± 530.35de	267.18 ± 24.20ef	0.66 ± 0.05a	0.77 ± 0.06de	1875.86 ± 221.36de	0.51 ± 0.08	1820.94 ± 445.82d	0.49 ± 0.07	79.96 ± 12.70abc
	60	54.56 ± 3.71b	87.07 ± 8.64f	3619.65 ± 518.80e	263.17 ± 23.71f	0.66 ± 0.04a	0.76 ± 0.04e	1806.40 ± 231.89e	0.50 ± 0.08	1813.26 ± 441.67d	0.50 ± 0.08	83.98 ± 12.93a
	67	56.28 ± 3.15a	90.56 ± 6.46de	3873.05 ± 403.73cd	274.26 ± 16.45de	0.65 ± 0.03a	0.77 ± 0.04de	1959.84 ± 339.07cde	0.50 ± 0.07	1913.21 ± 304.15bcd	0.50 ± 0.07	81.64 ± 10.61abc
	73	56.26 ± 3.27a	92.77 ± 5.95cd	3955.85 ± 388.52bc	281.48 ± 16.49cd	0.63 ± 0.03b	0.78 ± 0.04cd	1980.17 ± 381.22cd	0.49 ± 0.06	1975.67 ± 199.84abc	0.50 ± 0.06	81.25 ± 11.64abc
	80	56.74 ± 3.02a	95.42 ± 5.51bc	4115.07 ± 381.80ab	287.29 ± 16.55bc	0.63 ± 0.03b	0.79 ± 0.03bc	2108.13 ± 336.97abc	0.51 ± 0.05	2006.94 ± 189.52ab	0.49 ± 0.05	77.15 ± 9.95cd
	89	56.69 ± 3.01a	97.39 ± 6.16b	4195.00 ± 408.82a	294.84 ± 17.24ab	0.61 ± 0.03c	0.80 ± 0.03bc	2144.11 ± 358.09ab	0.51 ± 0.05	2050.89 ± 173.52a	0.49 ± 0.05	78.18 ± 10.87bcd
	102	55.89 ± 3.10ab	97.45 ± 6.08ab	4134.96 ± 388.70ab	293.22 ± 19.82ab	0.61 ± 0.04c	0.81 ± 0.03ab	2106.69 ± 358.44abc	0.50 ± 0.05	2028.27 ± 142.97ab	0.49 ± 0.05	83.29 ± 8.74ab
	110	55.77 ± 3.22ab	100.87 ± 7.96a	4277.89 ± 436.97a	300.66 ± 18.79a	0.60 ± 0.03c	0.82 ± 0.04a	2217.92 ± 370.89a	0.51 ± 0.05	2059.98 ± 179.84a	0.48 ± 0.05	79.55 ± 15.29abcd

"a–d" represent statistically significant differences at a significance level of 0.05.

As the reproductive period progressed, stomatal phenotypes showed a complex and orderly pattern of change. During the vegetative growth period, SD showed a significant change, and five stomatal size-related traits (SW, SL, SA, SP, SCA) and three stomatal shape traits (PSCA, GCPA) showed a decreasing trend. On the contrary, SD, SR, SE and PGCPA showed an increasing trend.

After entering the reproductive growth period, the trend of stomatal phenotypic traits changed significantly. SD gradually decreased, SR and PGCPA also showed a decreasing trend. On the contrary, five stomatal size-related traits (SW, SL, SA, SP, SCA) and three stomatal shape traits (SE, PSCA, GCPA) showed an increasing trend.

In the aging period, SD changed significantly at each sampling time point, indicating that the distribution of stomatal number changed significantly with leaf senescence and showed a trend of increasing and then decreasing. one stomatal size trait (SW) and two stomatal shape traits (SR and PGCPA) showed a trend of decreasing, one stomatal size trait (SL) and one stomatal shape trait (SE) showed a trend of increasing, while the remaining stomatal size traits (SA, SP, SCA, and GCPA) and stomatal shape trait (PGCPA) showed a trend of decreasing and then increasing trend.

### 3.4 Differential stomatal phenotypes in different leaf layers of maize

This study delved into the variability of stomatal characteristics of maize leaves from different leaf layers. The results showed that there were significant differences in several traits of stomata between different leaf layers (**Table 2**).

In the upper leaves, SD, GCPA, and 2 stomatal morphology traits (SE and PGCPA) were the highest values, indicating a greater SD and larger GCPA in the upper leaves. In the middle leaf layer, all three stomatal size traits (SL, SA and SP) were the highest values, indicating that the stomatal size of the middle leaf layer was larger relative to the other leaf layers; however, the stomatal shape indicator (SR) was the lowest value, indicating that the stomatal morphology of the middle leaf layer was narrower and longer relative to the other leaf layers, whereas in the lower leaf layer, the SW, SR, SCA and PSCA were all the highest values, indicating that the stomatal morphology of the lower leaves tended to be more rounded relative to the other leaf layers; the rest of the phenotypic traits were the lowest values among the leaf layers, indicating that the SD and stomatal size of the lower leaves were smaller relative to the other leaf layers.

### 3.5 Differential analysis of leaf stomatal phenotypes between hybrids and their parents

In this study, we synthesized stomatal data from different fertility periods and leaf layers and comparatively analyzed the differences in leaf stomatal phenotypes between hybrid 728 and its parents 2416 and MC01. The results showed that there were significant differences between the hybrid and its parents in several aspects of stomatal density, size and morphology (**Table 2**).

**Figure 4:**
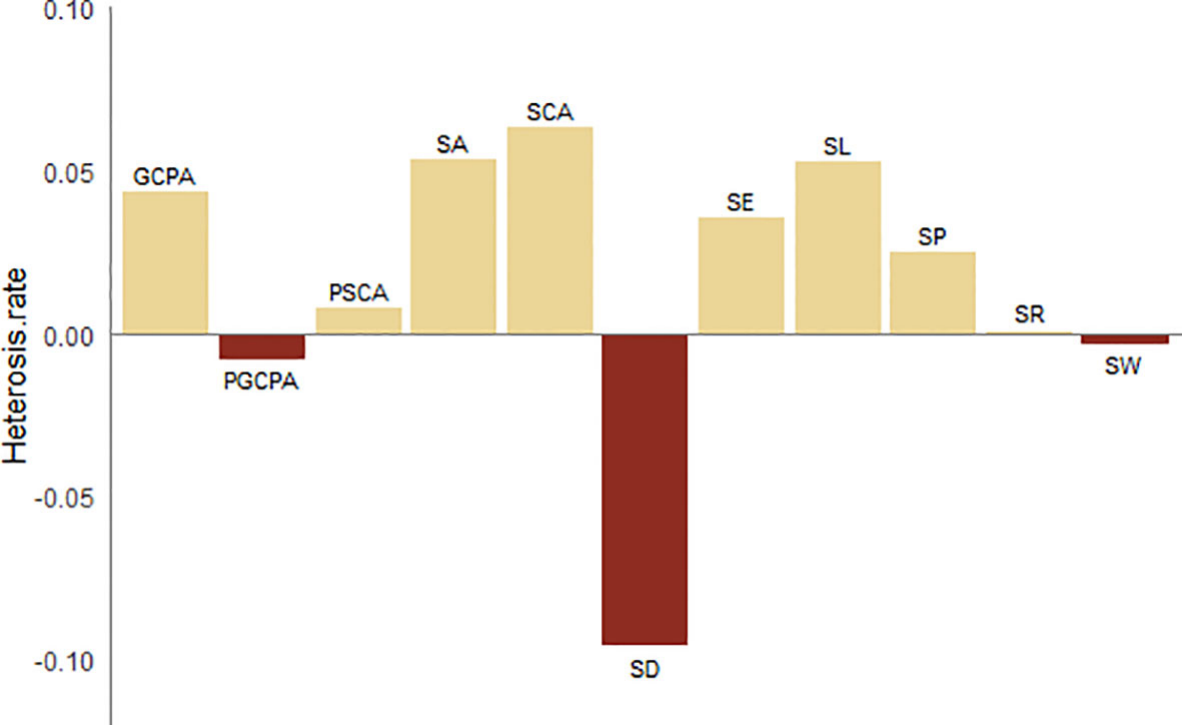
Heterosis rate of stomatal characters.

Overall, in terms of SD, parent 2416 had the highest SD, followed by parent MC01, while hybrid 728 showed a relatively low SD. This finding reveals that hybrids differ from their parents in stomatal distribution. In terms of stomatal size, SL, SA, SP, SCA and GCPA were the highest in hybrid 728, SW was more similar to the parent, and SA, SL, SCA and GCPA were more similar to the parent; and in terms of stomatal shape, SE was the highest in hybrid 728, and all the shape traits were more similar to the parent. **Figure 4** shows that the hybrids were negatively superparental for density and positively superparental for all size traits.

**Figure 5:**
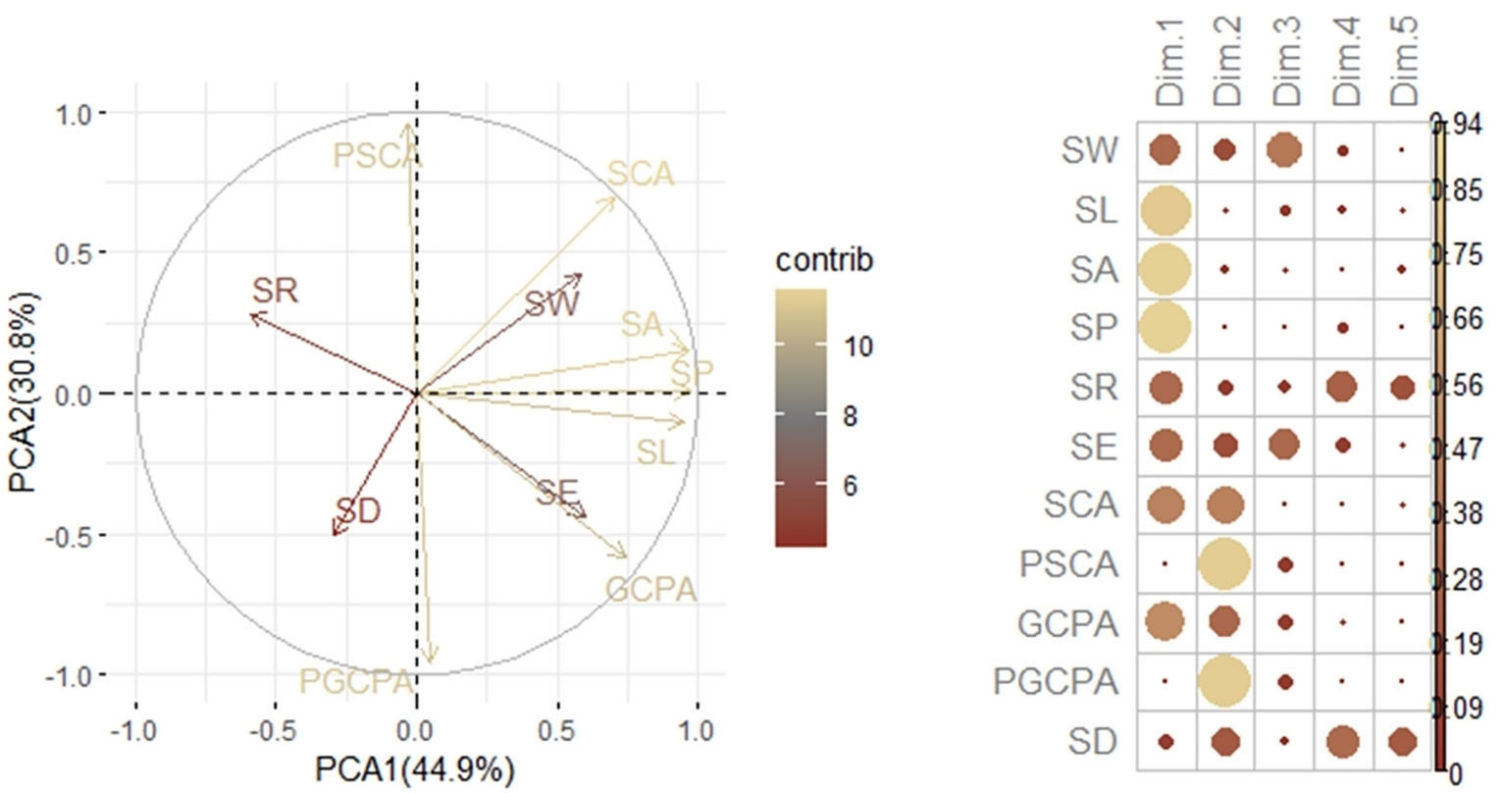
Principal component analysis of stomatal characters.

**Figure 6:**
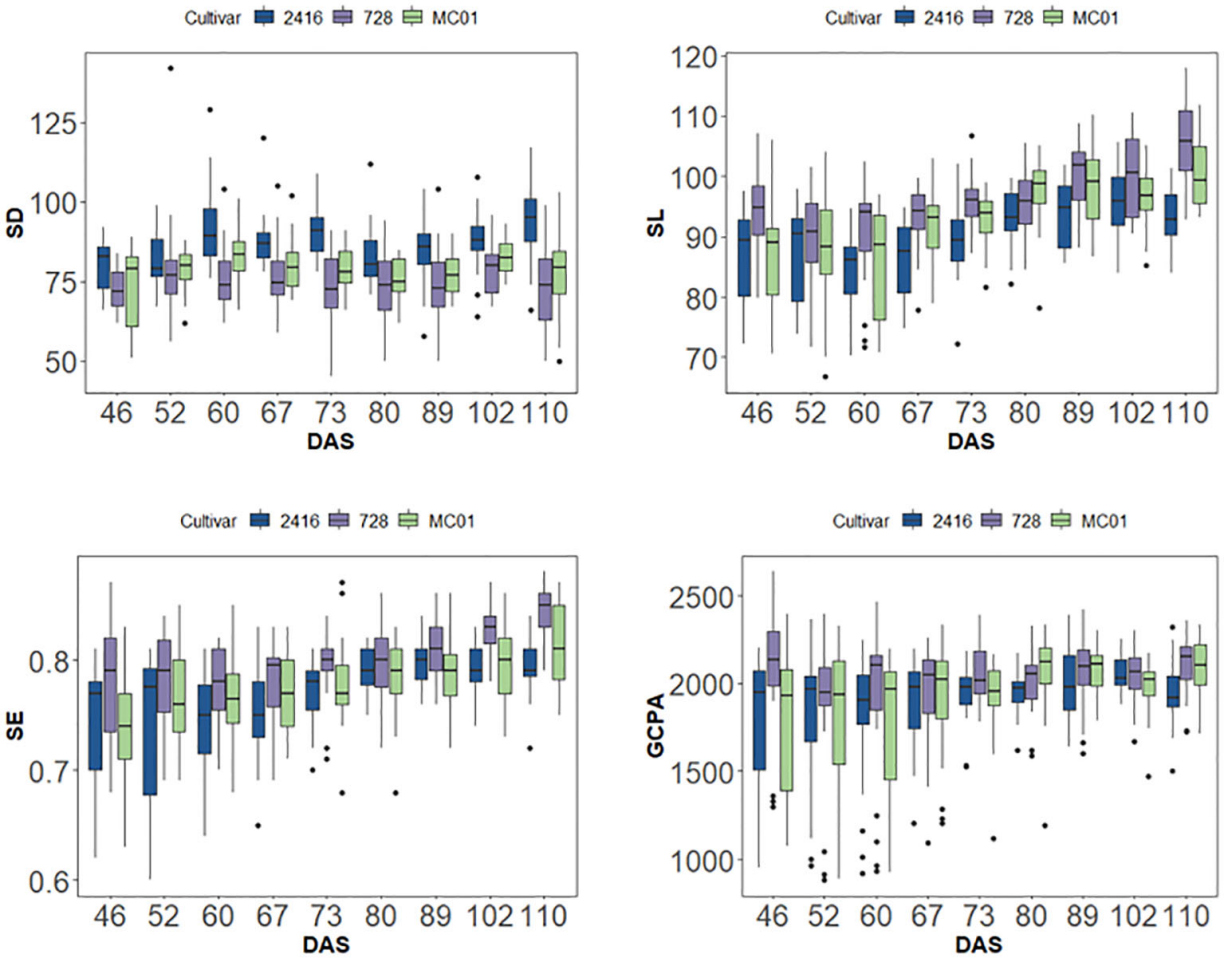
SD, SL, SE, PGCPA of different varieties in different periods.

During the reproductive process of maize, hybrid 728 and its parents also showed different stomatal phenotypic characteristics. As the reproductive process progressed, the differences in stomatal phenotypic traits between hybrid 728 and its parents 2416 and MC01 showed a decreasing and then increasing trend (**Table 3**). It is noteworthy that this difference reached the most significant level during the reproductive growth period. Based on the principal component analysis (**Figure 5**), four traits, SD, SL, SE, and PGCPA, were singled out for analysis, and it was found that hybrid 728 differed significantly from its parents during the reproductive growth period, and the trend was consistent with the overall trend (**Figure 6**; **Supplementary Figure 3**).

**Table 3:** Variance analysis of the effects of each cultivar and each period on the stomatal phenotypic characteristics of maize cultivars.

Cultivar	Time	SW	SL	SA	SP	SR	SE	SCA	SCA/SA	GCPA	GCPA/SA	SD
2416	46	56.27 ± 2.64	86.75 ± 8.80c	3688.76 ± 404.364bc	268.21 ± 27.80cd	0.65 ± 0.08a	0.74 ± 0.07b	1933.46 ± 303.89abc	0.53 ± 0.09	1755.30 ± 428.80c	0.47 ± 0.09	79.38 ± 9.22e
	52	55.29 ± 2.13	87.10 ± 7.95c	3657.32 ± 387.40bc	270.55 ± 20.43cd	0.64 ± 0.06ab	0.74 ± 0.07b	1867.59 ± 183.65abc	0.52 ± 0.08	1789.72 ± 417.86c	0.48 ± 0.08	81.75 ± 9.20de
	60	54.95 ± 2.53	84.75 ± 7.30c	3543.83 ± 379.36c	261.37 ± 19.23d	0.66 ± 0.04a	0.74 ± 0.05b	1745.53 ± 180.47c	0.50 ± 0.08	1798.30 ± 407.23bc	0.50 ± 0.08	92.56 ± 13.28ab
	67	55.52 ± 2.51	86.84 ± 6.27c	3653.13 ± 332.15cd	269.45 ± 16.12cd	0.64 ± 0.03ab	0.75 ± 0.05b	1778.35 ± 244.48bc	0.49 ± 0.06	1874.78 ± 272.39abc	0.51 ± 0.06	88.22 ± 9.41abcd
	73	55.55 ± 2.04	89.59 ± 6.69bc	3760.57 ± 403.04bc	280.25 ± 18.25bc	0.61 ± 0.03bc	0.77 ± 0.03ab	1821.10 ± 341.51abc	0.48 ± 0.05	1939.48 ± 171.46abc	0.52 ± 0.05	90.74 ± 8.89abc
	80	55.37 ± 1.84	93.23 ± 4.64ab	3915.03 ± 278.74ab	287.91 ± 18.36ab	0.60 ± 0.04c	0.79 ± 0.02a	1963.94 ± 255.76ab	0.50 ± 0.04	1951.09 ± 126.28abc	0.50 ± 0.04	83.20 ± 9.65cde
	89	54.95 ± 2.15	93.91 ± 5.42ab	3906.98 ± 331.11ab	290.40 ± 20.29ab	0.59 ± 0.04c	0.80 ± 0.02a	1900.53 ± 257.87abc	0.48 ± 0.04	2006.44 ± 187.26ab	0.52 ± 0.04	84.23 ± 10.04bcde
	102	56.45 ± 2.13	95.73 ± 5.48a	4087.42 ± 335.82a	300.18 ± 22.86a	0.58 ± 0.04c	0.79 ± 0.02a	2027.74 ± 304.52a	0.49 ± 0.04	2059.67 ± 107.63a	0.51 ± 0.04	88.22 ± 9.28abcd
	110	55.02 ± 2.23	93.00 ± 4.43ab	3877.25 ± 253.47ab	288.42 ± 13.36ab	0.59 ± 0.03c	0.79 ± 0.03a	1934.00 ± 211.14abc	0.50 ± 0.04	1943.24 ± 178.52abc	0.50 ± 0.04	93.87 ± 11.67a
MC01	46	57.00 ± 5.12ab	87.17 ± 9.08de	3778.74 ± 597.17cde	267.01 ± 27.41def	0.67 ± 0.05a	0.74 ± 0.05c	1995.65 ± 290.47cde	0.53 ± 0.07	1783.09 ± 455.21b	0.47 ± 0.07	71.54 ± 14.44c
	52	54.40 ± 4.01bc	87.97 ± 11.16de	3660.24 ± 635.63de	262.36 ± 25.44ef	0.67 ± 0.03a	0.77 ± 0.05bc	1858.97 ± 228.28de	0.51 ± 0.06	1801.27 ± 480.83b	0.49 ± 0.07	78.20 ± 7.56abc
	60	53.32 ± 4.30c	86.03 ± 9.10e	3487.52 ± 559.24e	257.18 ± 24.62f	0.67 ± 0.04ab	0.77 ± 0.04bc	1739.08 ± 200.30e	0.50 ± 0.08	1748.44 ± 461.53b	0.50 ± 0.08	83.89 ± 8.98a
	67	56.53 ± 3.59ab	91.15 ± 6.32cde	3910.95 ± 440.03cd	274.46 ± 18.25cde	0.66 ± 0.03ab	0.77 ± 0.04bc	2012.02 ± 338.40bcde	0.51 ± 0.07	1898.93 ± 345.81ab	0.49 ± 0.07	79.95 ± 8.46ab
	73	56.59 ± 4.42ab	92.90 ± 4.64bcd	3977.98 ± 359.94bcd	278.79 ± 17.75bcd	0.65 ± 0.04abc	0.78 ± 0.04ab	2046.44 ± 356.96abcd	0.51 ± 0.07	1931.54 ± 242.81ab	0.49 ± 0.06	79.74 ± 7.38ab
	80	58.64 ± 2.93a	97.31 ± 5.93ab	4330.42 ± 409.55ab	291.00 ± 15.73ab	0.65 ± 0.02bc	0.78 ± 0.03ab	2274.27 ± 330.35ab	0.52 ± 0.05	2056.16 ± 236.78a	0.48 ± 0.05	76.05 ± 6.35bc
	89	58.49 ± 3.21a	98.34 ± 6.13ab	4361.63 ± 368.95a	296.43 ± 15.23a	0.63 ± 0.02cd	0.79 ± 0.04ab	2279.19 ± 393.78ab	0.52 ± 0.05	2082.45 ± 122.06a	0.48 ± 0.05	77.38 ± 6.62abc
	102	56.49 ± 3.81abc	96.68 ± 4.87abc	4150.22 ± 358.65abc	285.50 ± 12.60abc	0.65 ± 0.02bc	0.80 ± 0.04ab	2170.87 ± 333.79abc	0.52 ± 0.05	1979.35 ± 159.71ab	0.48 ± 0.04	82.82 ± 6.18ab
	110	57.12 ± 4.26ab	101.10 ± 6.14a	4385.87 ± 420.54a	300.03 ± 15.58a	0.62 ± 0.03d	0.81 ± 0.04a	2298.76 ± 380.98a	0.52 ± 0.05	2087.11 ± 165.71a	0.48 ± 0.05	76.96 ± 13.36abc
728	46	57.43 ± 3.83a	94.71 ± 7.51bcd	4125.44 ± 390.75bc	288.38 ± 14.97bc	0.63 ± 0.04bcd	0.78 ± 0.06d	2072.15 ± 329.93bc	0.50 ± 0.08	2053.29 ± 414.29ab	0.49 ± 0.08	72.60 ± 6.24
	52	54.11 ± 3.74c	90.34 ± 9.17cd	3762.37 ± 567.29d	268.20 ± 26.84e	0.66 ± 0.05a	0.79 ± 0.04cd	1897.29 ± 254.70c	0.50 ± 0.08	1865.09 ± 461.96b	0.49 ± 0.08	79.78 ± 18.16
	60	55.36 ± 3.92abc	90.26 ± 8.83d	3816.67 ± 558.55cd	270.55 ± 25.84de	0.66 ± 0.04a	0.78 ± 0.04d	1927.84 ± 262.43c	0.51 ± 0.08	1888.83 ± 465.78b	0.49 ± 0.08	76.00 ± 11.06
	67	56.71 ± 3.24ab	93.31 ± 5.36cd	4033.09 ± 349.81bcd	278.40 ± 14.35cde	0.66 ± 0.02ab	0.78 ± 0.04d	2071.01 ± 360.32bc	0.51 ± 0.07	1962.08 ± 295.68ab	0.49 ± 0.07	77.55 ± 11.13
	73	56.63 ± 2.98ab	95.66 ± 4.94bc	4120.33 ± 330.34bc	285.21 ± 13.36bcd	0.64 ± 0.02abc	0.80 ± 0.03cd	2068.35 ± 408.53bc	0.50 ± 0.06	2051.98 ± 163.45ab	0.50 ± 0.06	73.80 ± 11.43
	80	56.20 ± 3.16abc	95.58 ± 5.33bcd	4092.39 ± 341.57bc	283.37 ± 15.46bcd	0.65 ± 0.02ab	0.80 ± 0.03cd	2081.82 ± 351.42abc	0.50 ± 0.06	2010.57 ± 181.52ab	0.49 ± 0.06	72.87 ± 10.75
	89	56.77 ± 2.62ab	99.99 ± 5.43b	4329.21 ± 367.24ab	297.82 ± 15.30ab	0.62 ± 0.02cd	0.81 ± 0.02bc	2262.99 ± 285.69ab	0.52 ± 0.04	2066.21 ± 151.15ab	0.48 ± 0.04	73.08 ± 12.30
	102	54.76 ± 2.98bc	99.90 ± 7.06b	4167.91 ± 469.95ab	293.64 ± 20.30b	0.61 ± 0.02de	0.83 ± 0.02ab	2124.24 ± 425.73abc	0.50 ± 0.06	2043.67 ± 151.15ab	0.50 ± 0.05	78.96 ± 7.95
	110	55.25 ± 2.57abc	105.88 ± 6.83a	4460.96 ± 377.50a	309.16 ± 19.79a	0.59 ± 0.03e	0.84 ± 0.02a	2344.44 ± 352.47a	0.52 ± 0.05	2116.53 ± 158.33a	0.48 ± 0.05	72.31 ± 12.57

"a–d" represent statistically significant differences at a significance level of 0.05.

**Figure 7:**
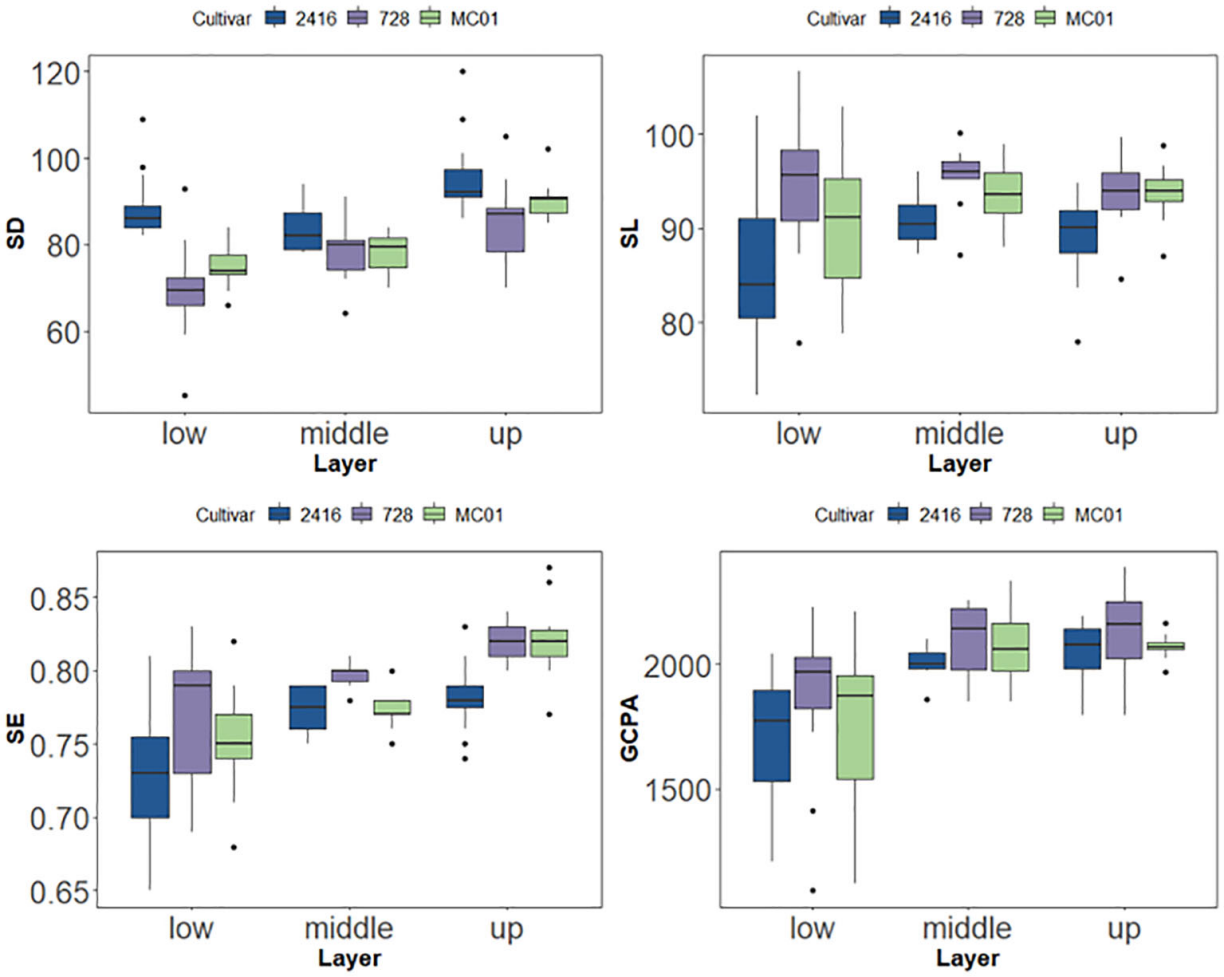
SD、SL、SE and GCPA of tested cultivars under different layer.

The results of stomatal phenotypic characterization showed that the phenotypic characteristics of hybrid 728 and its parents did not change significantly over time between 67 and 73 days after sowing. Based on this stability, the present study was conducted to analyze the stomatal phenotypes of different leaf layers for hybrid dominance based on data from this period (**Table 2**). The results showed that there were significant differences in stomatal phenotypes between hybrid 728 and its parents in different leaf layers (**Table 4**; **Supplementary Figure 4**), and hybrid 728 was superior to its parents in the four phenotypic traits of SL, SE, GCPA, and SD, and the hybrid had a greater SL and PGCPA, higher SE, and a smaller SD in all leaf layers compared with its parents (**Figure 7**). The uniqueness and complexity of stomatal characteristics of the hybrids were further revealed.

**Table 4:** Variance analysis of the effect of each leaf layer of each cultivar on the stomatal phenotypic characteristics of maize cultivars.

Cultivar	Layer	SW	SL	SA	SP	SR	SE	SCA	PSCA	GCPA	GCPA/SA	SD
2416	low	56.22 ± 2.65a	85.80 ± 8.73	3656.80 ± 511.45	274.16 ± 23.94	0.62 ± 0.04	0.73 ± 0.05a	1931.56 ± 367.23a	0.52 ± 0.05a	1902.461 ± 275.951b	0.490 ± 0.0533a	88 ± 12a
	middle	56.30 ± 1.06a	90.90 ± 2.62	3847.45 ± 158.59	282.07 ± 10.58	0.61 ± 0.03	0.77 ± 0.02b	1842.57 ± 164.39ab	0.48 ± 0.03b	1975.688 ± 321.815a	0.467 ± 0.0584b	74 ± 12c
	up	54.07 ± 1.78b	89.12 ± 4.73	3656.73 ± 261.51	270.15 ± 12.32	0.63 ± 0.02	0.78 ± 0.02b	1601.00 ± 150.18b	0.43 ± 0.01c	1927.320 ± 334.075ab	0.461 ± 0.0561b	78 ± 10b
MC01	low	58.04 ± 3.30a	90.15 ± 6.82	3960.05 ± 467.09ab	279.13 ± 21.80	0.65 ± 0.04	0.75 ± 0.03b	2213.86 ± 291.67a	0.56 ± 0.06a	1782.836 ± 359.614b	0.441 ± 0.0557c	75 ± 12c
	middle	58.35 ± 2.70a	93.83 ± 3.68	4148.56 ± 325.68a	280.97 ± 15.15	0.67 ± 0.02	0.77 ± 0.01b	2070.02 ± 261.21a	0.49 ± 0.04b	2067.823 ± 165.433a	0.486 ± 0.0355b	81 ± 11b
	up	51.95 ± 2.33b	93.70 ± 3.20	3707.39 ± 145.24b	267.29 ± 6.52	0.66 ± 0.03	0.82 ± 0.03a	1635.92 ± 117.48b	0.44 ± 0.02c	2083.650 ± 177.192a	0.512 ± 0.0439a	86 ± 12a
728	low	58.76 ± 1.60a	94.31 ± 6.39	4209.04 ± 315.63a	286.13 ± 13.54a	0.65 ± 0.03	0.77 ± 0.04b	2313.69 ± 301.70a	0.55 ± 0.06a	2014.151 ± 214.680a	0.472 ± 0.0381b	81 ± 10ab
	middle	56.53 ± 1.44b	95.47 ± 3.51	4110.61 ± 240.18a	283.36 ± 11.27ab	0.65 ± 0.02	0.80 ± 0.01ab	2010.36 ± 267.51b	0.48 ± 0.05b	2015.467 ± 202.392a	0.472 ± 0.0367b	77 ± 16c
	up	52.65 ± 2.42c	93.85 ± 4.26	3778.13 ± 299.87b	271.61 ± 13.76b	0.65 ± 0.02	0.82 ± 0.01a	1640.98 ± 149.29c	0.43 ± 0.02b	1893.317 ± 366.078bc	0.502 ± 0.0770a	81 ± 15ab

"a–d" represent statistically significant differences at a significance level of 0.05.

## 4 Discussion

The means of stomatal phenotype acquisition has gone through a long stage of development, and is currently divided into two categories of destructive and non-destructive methods. In this experiment, we used the fresh sample method of destructive sampling methods, which is more time-saving and labor-saving, and also utilized the rapid scanning electron microscope (RSEM), which eliminates the cumbersome sample pre-processing, and acquires more accurate and clearer image information. The leaf stomatal phenotype was finely segmented using target detection and deep learning, and the target detection model based on YOLOV5s and the UNet semantic segmentation model were developed and integrated to accurately parse the stomatal phenotype,YOLOv5s is a lightweight target detection model with fast processing speed and high accuracy. While Unet is a deep learning model commonly used for image segmentation, the single image processing time for stomatal number resolution based on the YOLOv5s model is about 0.009s/sheet, and the detection efficiency for stomatal depth trait resolution based on the UNet model is 0.815s/sheet on average. By combining these two models, efficient detection and accurate segmentation of stomatal targets were realized, so that several phenotypic indicators, including stomatal number, shape, distribution, etc., could be extracted from the complex background, which is much improved over the traditional manual extraction of stomatal phenotypes, saves manpower, can quickly process a large amount of image data, improves the efficiency of stomatal phenotypic feature extraction, and has a better segmentation effect. In addition to obtaining the traditional stomatal phenotype traits, this experiment also obtained four traits related to stomatal subsidiary cells and stomatal guard cells and pores. With the continuous development of machine learning, target detection and cell segmentation are classical tasks in biological image processing, and many practical tools have been constructed for different scenarios, such as ITK morphological watershed (McCormick et al., 2014), MorphoGraphX (Barbier De Reuille et al., 2015), PaCeQuant (Möller et al., 2017), StomataCounter (Fetter et al., 2019), PlantSeg (Wolny et al., 2020), Cellpose (Stringer et al., 2021) and LeafNet (Li et al., 2022). Reasonable utilization of these software and improvement of the network model can be used to mine the image information in a deeper level and solve the scientific research problems.

The phenotypic results of maize leaf stomata related to stomatal density, size and shape on the leaf layer were basically in agreement with the results of previous studies (Zhao et al., 2001; Wang et al., 2004), with a significant negative correlation between stomatal number and stomatal size. In general, an increase in light intensity leads to an increase in stomatal index (Lake et al., 2001; Casson and Gray, 2008). In the upper leaves, SD and GCPA were larger and more helpful for effective gas exchange in the upper leaf. Lower stomata had higher SW and SR but the lowest values of other phenotypic traits (SD, GCPA, SE, etc.), indicating that lower stomata were smaller in size but more round in shape. Stomatal density increased significantly with increasing light intensity (Fan et al., 2013). Smaller SS coupled with higher SD usually leads to higher Gsmax, and smaller stomatal size can reduce the total pore area of the leaf and may also promote a faster pore size response (Franks et al., 2009; Drake et al., 2013; Lawson and Blatt, 2014). The higher the cell surface area to volume ratio of smaller cells, the faster the ion flux, which leads to faster changes in guard cell expansion and a more rapid Gs response (Lawson and Vialet-Chabrand, 2019). Upper leaf stomatal characteristics respond well to these conditions. the three size-related traits (SL, SA, SP) were the highest, but SR was the smallest, indicating that stomata were larger but more narrowly shaped relative to the other leaf layers. Corn leaf stomata are constantly changing during growth, and when senescence occurs, leaf water content is low and stomatal density increases. As leaf relative water content (RWC) decreases, stomatal conductance (g(s)) gradually decreases and the rate of CO_2_ assimilation (A) slows down and eventually stops (Lawlor, 2002). Among them, stomata were more stable at 67-73d after sowing, which was closely related to plant growth status. During this time period the maize plant has reached its maximum plant size and is in the spitting stage. Some research leaves believe that the leaf vein state is more stable during the period of time when maize reaches its maximum plant size.

The hybrids were reduced in stomatal density, larger in size, narrower and more elongated in morphology, and had larger guard cells and pore areas than the parents. It has been shown that the maximum photosynthetic rate of the offspring was significantly higher than that of the parents at the same leaf position, it indicated that the offspring has stronger photosynthetic capacity, which can optimize the CO_2_ absorption of photosynthesis and improve the efficiency of photosynthesis, and the increase in photosynthetic rate helps the plant to accumulate more organic matter and provide sufficient energy and material base for growth and development; SPAD was significantly affected by variety, with the offspring 728 being the largest, the parent 2416 being the second largest, and the mother MC01 being the smallest. Generally, crops optimize their own configuration to achieve appropriate growth targets (Franks and Beerling, 2009). Bertolino et al. (2019) found that by changing the pore size of stomata, plants were able to optimize CO_2_ uptake for photosynthesis while minimizing water loss, and he suggested that a reduction in the number of stomata by the plant may be an effective water use efficiency and drought tolerance of the plant without decreasing the yield means. The significant reduction in SD resulted in increased plant tolerance to drought without adverse effects on nitrogen and phosphorus uptake (Hepworth et al., 2015, 2016). It has been suggested that increased leaf carbohydrate-related photosynthetic efficiency may contribute to higher growth rate, biomass and yield in F1 hybrid progeny (Meena et al., 2021), while hybrids with larger guard cells have improved photosynthetic efficiency. Khazaie, H (Khazaie et al., 2011), it was found that in different genotypes of wheat, stomatal frequency was negatively correlated with seed yield, and stomatal area was closely and positively correlated with seed yield. The abaxial leaf stomatal frequency had a negative and significant indirect effect on leaf photosynthetic rate. Stomatal area also had a significant indirect effect on photosynthetic rate by carbon isotope discrimination. The hybrids were characterized on leaf stomata as an optimization of maize growth, showing where the advantages lie on a microscopic scale. It has been found that drought leads to a significant increase in stomatal density, a decrease in stomatal size, and a decrease in transpiration rate; therefore, small leaf stomatal density and large size are beneficial to reduce water loss (Caine et al., 2019; Dunn et al., 2019), and can improve water utilization and drought tolerance. Hybrid 728 promotes water-air exchange and improves drought tolerance in maize more than the parents.

Climate change in recent years, including elevated carbon dioxide levels, high temperatures and droughts, has had a major impact on the ecosystem structure and productivity of global agriculture. Water scarcity is one of the important factors constraining the development of agriculture and national economy in China, and improving the efficiency of plant water utilization is a major strategic issue for food production in the world. Stomata are the main channels for gas exchange between plant leaves and the outside world, and almost all of the water required for plant growth is lost to the atmosphere through stomatal transpiration. In this study, we investigated the stomata of the whole unfolded leaf blades of three maize species at the whole reproductive stage, revealing the general pattern of stomatal status of maize plants and the differences between hybrids and their parents, and made a preliminary exploration of stomatal hybrid dominance, provides a new idea for the accurate identification of stomatal phenotypes and supports the genetic analysis and targeted improvement of maize stomatal traits. It is of great significance for improving crop yield, drought resistance and water utilization efficiency, and is conducive to screening high-quality, high-yielding and drought-resistant varieties, providing a basis for new variety selection and breeding. The test method can also be applied to other varieties. More materials are needed to verify whether the conclusions are applicable to other maize varieties.

## 5 Conclusion

**Figure 8:**
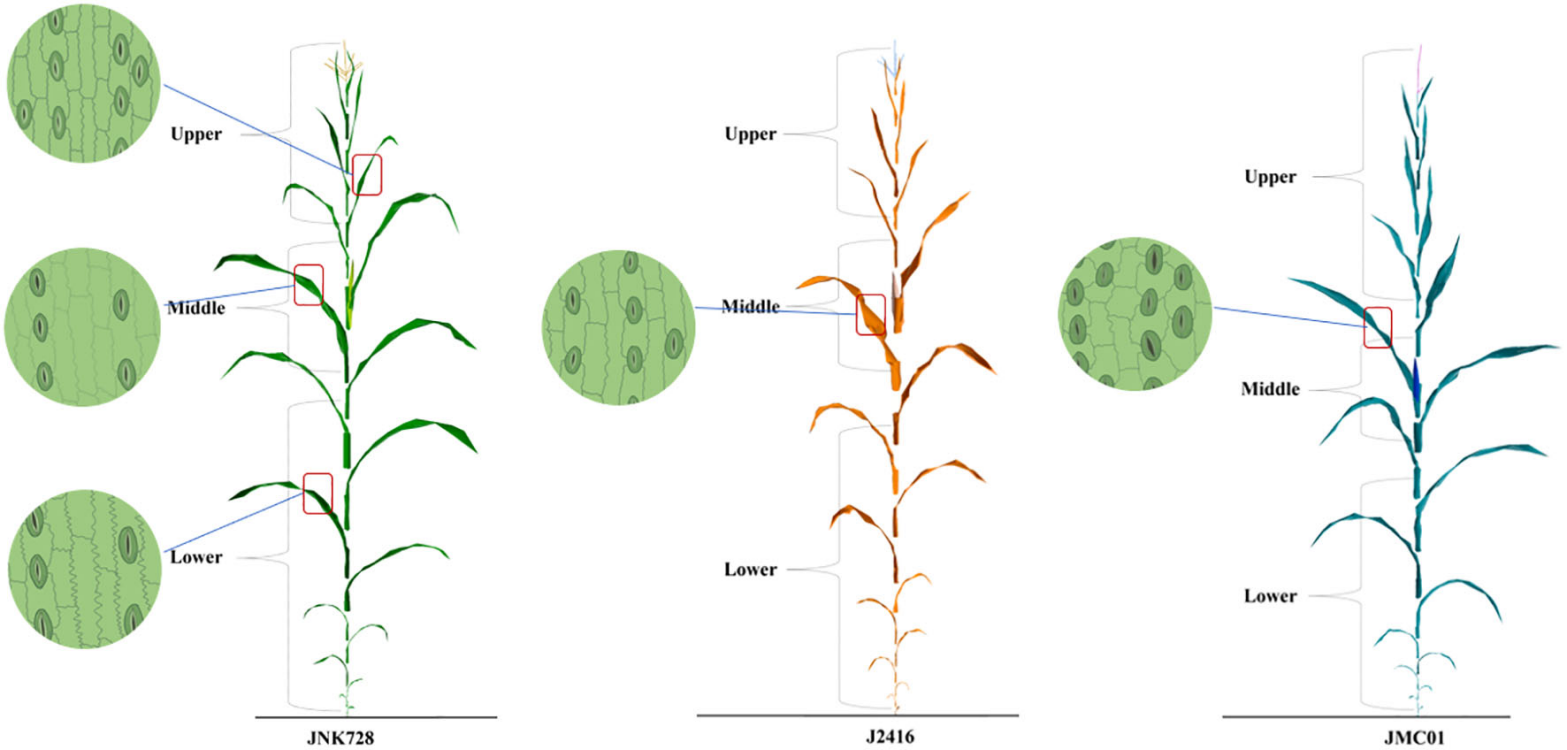
Schematic representation of stomata of each cultivar.

In this study, the changing pattern of stomatal phenotypes in the lower epidermis of maize leaves at different reproductive stages was investigated in depth using Jingnongke 728 and its parents. The results showed (**Figure 8**) that at the single-plant scale, stomatal density showed an increasing trend from bottom to top, and the shape shifted from subcircular to narrow and elongated, with the size being the largest in the middle layer. At the same time, the percentage of guard cells and pore area was also characterized by the upper layer being larger than the middle layer, the middle layer being larger than the lower layer, and the lower layer had the smallest guard cells and pore area.

In terms of temporal changes, stomatal density remained stable during the vegetative growth period, while stomatal size gradually decreased; after entering the reproductive growth period, stomatal density and size decreased, and the shape became narrower and more elongated. At the aging period, stomatal density continued to decrease, while stomatal size increased and the shape tended to be rounded. It is worth noting that hybrid 728 showed significant differences in stomatal phenotype from its parents: its stomatal density was lower, its size was larger, and its guard cells and pores were larger, closer to the parent MC01, and more significantly different from the parent 2416. The differences in stomatal phenotypes among the three varieties gradually widened as fertility progressed, reaching a maximum at senescence.

This study not only realized the high-throughput acquisition and precise analysis of stomatal phenotypes in the whole folded leaves of maize hybrids, but also preliminarily revealed the dynamic change patterns of the phenotypic indexes in different fertility periods and leaf layers. Stomatal hybrid dominance is of great significance in enhancing crop yield, improving drought resistance and water utilization efficiency, which is conducive to the selection of high-quality, high-yielding and drought-tolerant varieties. These findings provide valuable references for the genetic analysis of maize leaf stomatal phenotypes and the targeted improvement of traits, which will help to deepen our understanding of maize stomatal development and functional characteristics, and provide new ideas and methods for maize high-yield and high-quality breeding.

## Data Availability

The original contributions presented in the study are included in the article/**Supplementary Material**. Further inquiries can be directed to the corresponding authors.
