# Supplementary material for: Analysis of stomatal characteristics of maize hybrids and their parental inbred lines during critical reproductive periods
Source: Front Plant Sci. 2025 Jan 16;15:1442686. doi: 10.3389/fpls.2024.1442686 (PMC11779725; doi:10.3389/fpls.2024.1442686)
Supplement: Supplementary file 1 [file DataSheet1.docx]

**Supplementary Materials**


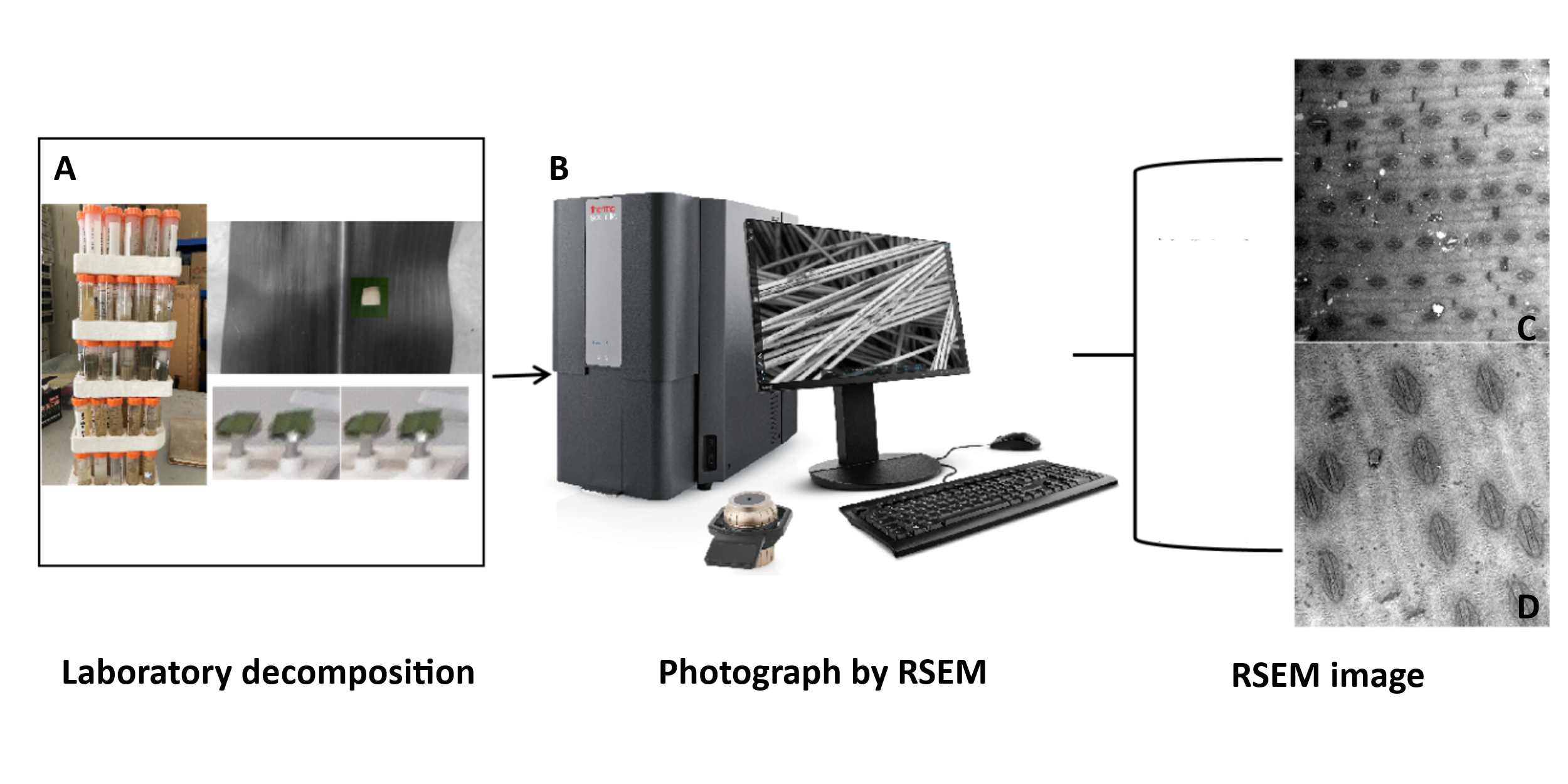


**Supplementary Figure 1 Process of stomatal image acquisition**

Note: The main process of stomatal image acquisition contains preparing leaves in the lab and taking pictures by RSEM


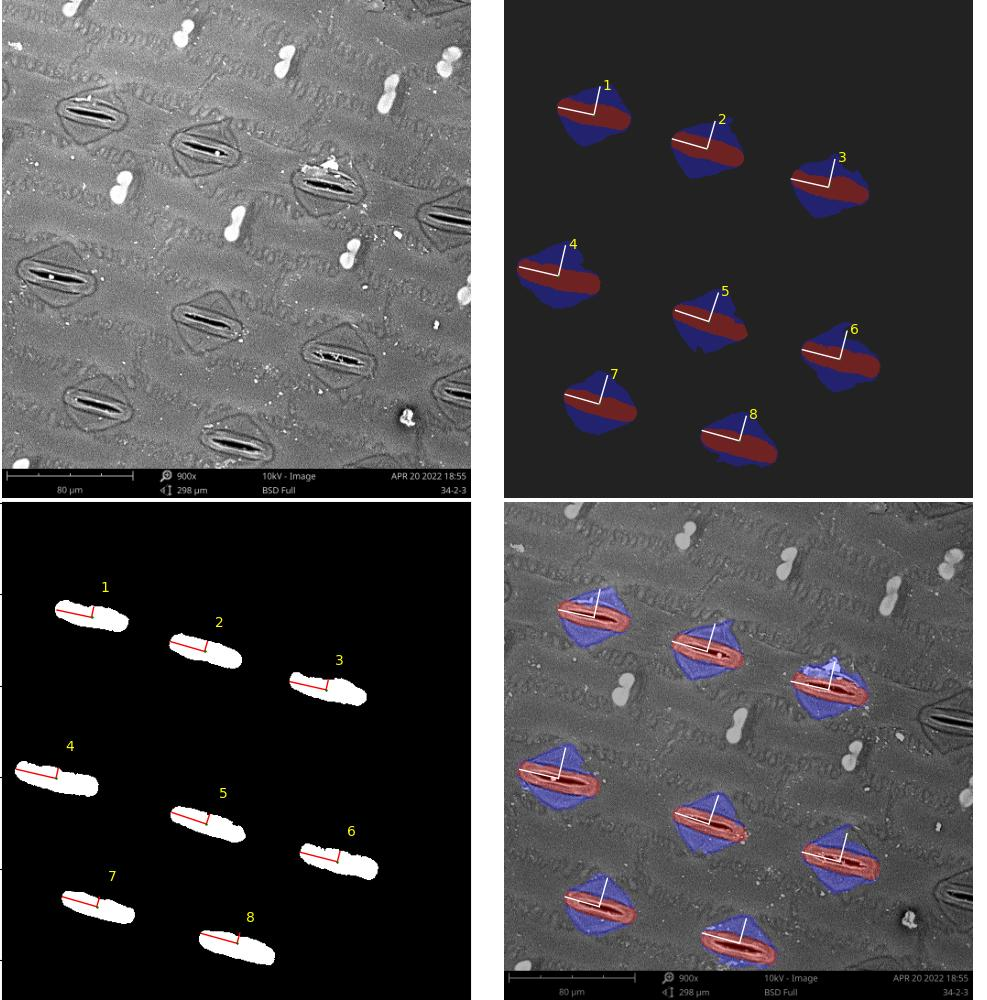


**Supplementary Figure 2 Stomatal segmentation by UNet**


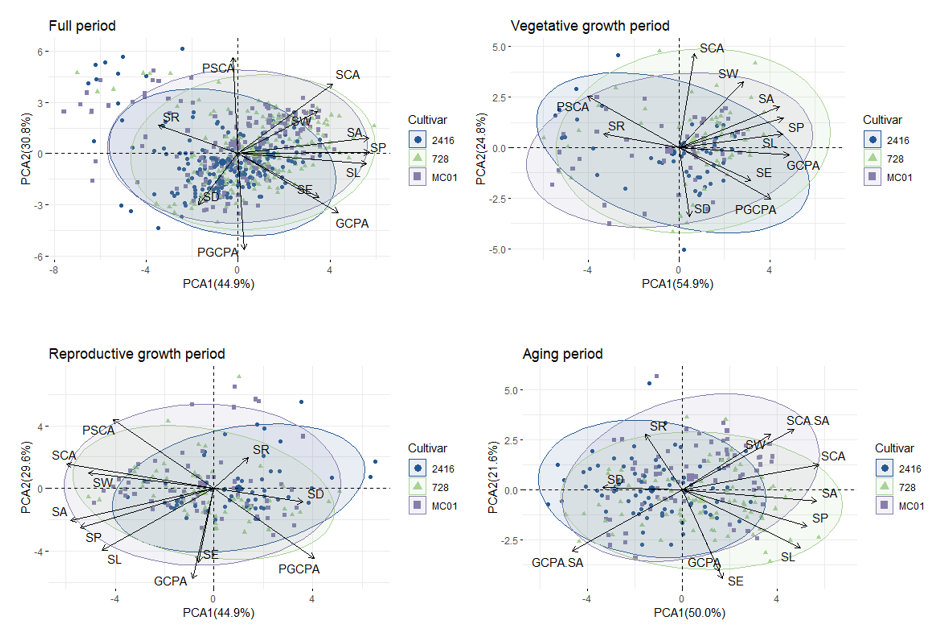


**Supplementary Figure 3** **Principal component analysis of stomatal traits in cultivars at different periods**


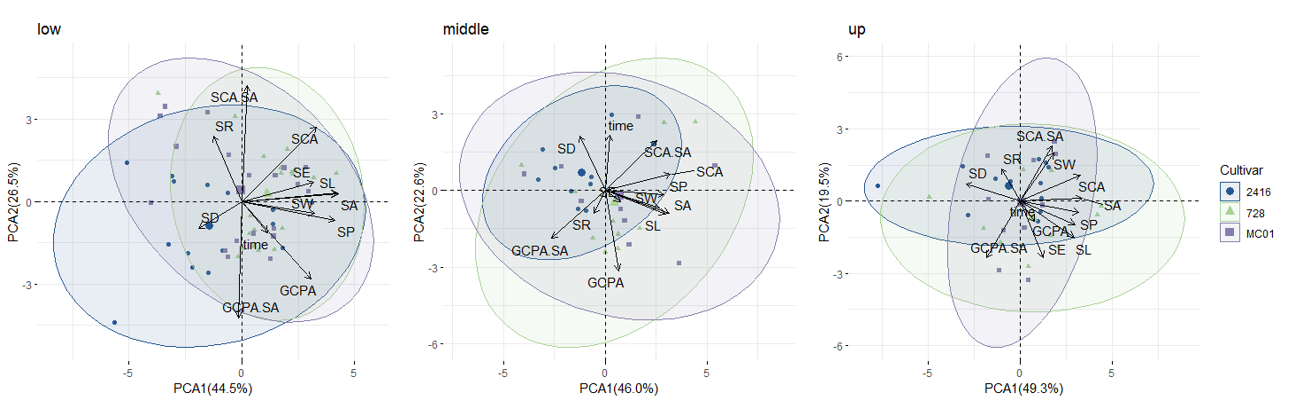


**Supplementary Figure 4 Principal component analysis of stomatal traits in cultivars at different layers of 67-73 days**
